# Speech loss in children with epilepsy: Not always Landau–Kleffner syndrome

**DOI:** 10.1111/dmcn.16108

**Published:** 2024-10-04

**Authors:** Patrick Van Bogaert

**Affiliations:** ^1^ Department of Pediatric Neurology CHU d'Angers Angers France

## Abstract

This commentary is on the original article by Eyre et al. on pages 392–404 of this issue.

Language regression in the context of childhood epilepsy often raises the hypothesis of a disorder that belongs to the epilepsy‐aphasia spectrum. This spectrum encompasses Landau–Kleffner syndrome (LKS), which was included among the developmental and/or epileptic encephalopathies with spike–wave activation in sleep (D/EE‐SWAS) in the last classification of epilepsy syndromes of childhood and milder forms that are frontier forms between D/EE‐SWAS and self‐limited epilepsy with centrotemporal spikes. In LKS, speech dysfunction is foremost receptive and classically consists of auditory verbal agnosia (i.e. the impossibility to give a semantic meaning to the phonemes), leading to a severe expressive and receptive verbal deficit. Studies have shown that genomic alterations of the NMDA glutamate receptor subunit gene *GRIN2A* are a major cause of LKS.^1^

Eyre et al. have highlighted that some children with epilepsy with language regression may present a purely or predominantly motor (expressive) speech dysfunction, with a variable combination of verbal dyspraxia and dysarthria.^2^ About half of their cohort of 18 children were diagnosed with D/EE‐SWAS, some of them carrying a pathogenic *GRIN2A* variant. This particular presentation of D/EE‐SWAS with deterioration of oral motor function and preserved receptive language has previously been reported under acquired epileptic opercular syndrome or drooling and oromotor dyspraxia in benign childhood epilepsy with centrotemporal spikes, eventually associated with a *GRIN2A* gene variant. This stresses the fact that the *GRIN2A* gene can be associated with both purely expressive and mixed language regressions in the context of D/EE‐SWAS.^3^, ^4^

Another half of the cohort Eyre et al. studied had Rasmussen syndrome. As expected, the outcome of the speech disorder strongly differed between the two groups. Patients with Rasmussen syndrome showed progressive worsening of speech, emphasizing that the existing medical therapeutic approaches for Rasmussen syndrome remain very disappointing. By contrast, speech tended to improve in patients with D/EE‐SWAS and even normalized in some of them, including one patient carrying a *GRIN2A* mutation. This is in line with the concept of epileptic encephalopathy, a condition in which the epileptiform abnormalities are believed to contribute to the disturbance in cerebral function. Indeed, it seems likely that speech improvement was related to the disappearance of intense spiking activity during sleep following appropriate antiepileptic treatment. Unfortunately, the relationship between electroencephalogram and clinical changes was not precisely studied in the paper by Eyre et al. This is a weakness of the paper as previous observations of patients with LKS have shown that the clinical evolution of the language dysfunction does not always strictly fit with electroencephalograph findings.^5^

Finally, Eyre et al. stress that in many of their patients rehabilitation was not provided by a speech and language therapist. This is surprising as these children are extremely disabled by their speech disorder and are prone to adopt non‐verbal strategies of communication, considering their preserved non‐verbal and receptive verbal skills. This paper should help to highlight the spectrum of speech and language dysfunction that may occur in association with epilepsy in childhood. It will also hopefully improve the dialogue between speech and language therapists and physicians in order to achieve a timely and appropriate work‐up, and offer the most suitable therapeutic strategy for speech and language regression in a child with epilepsy.

## Data Availability

Not required.
